# Successful intraoperative radiation therapy for a rapidly recurrent smooth muscle tumor of uncertain malignant potential

**DOI:** 10.1002/kjm2.12831

**Published:** 2024-04-16

**Authors:** Yu‐Hsuan Chung, Peir‐In Liang, Feng‐Hsiang Tang

**Affiliations:** ^1^ School of Medicine, College of Medicine, Kaohsiung Medical University Kaohsiung Taiwan; ^2^ Department of Pathology Kaohsiung Medical University Hospital, Kaohsiung Medical University Kaohsiung Taiwan; ^3^ Department of Obstetrics and Gynecology Kaohsiung Municipal Ta‐Tung Hospital, Kaohsiung Medical University Kaohsiung Taiwan; ^4^ Department of Obstetrics and Gynecology Kaohsiung Medical University Hospital, Kaohsiung Medical University Kaohsiung Taiwan

Smooth muscle tumors of uncertain malignant potential (STUMPs) represent a group of rare uterine neoplasms whose morphological features are between those of leiomyoma and leiomyosarcoma.^1^ STUMP recurrence poses a major challenge because no gold standard for treatment has been established. The present case highlights the effective use of intraoperative radiotherapy (IORT) as a salvage technique in a patient with twice recurrent STUMP, with the treatment resulting in more than 5 years of disease‐free survival.

A 46‐year‐old woman presented with large myoma and compression symptoms and underwent laparoscopic myomectomy. Her pathology report revealed STUMPs requiring laparoscopic hysterectomy. Six months postoperation, the patient visited our outpatient department for dull intermittent right‐lower‐quadrant abdominal pain. Laboratory data revealed elevated C‐reactive protein (58.5 mg/dL) and CA‐125 levels (Figure 1A). Transabdominal sonography revealed a right adnexal mass measuring 5.44 × 5.29 cm^2^ with complex echogenicity. Abdominal computed tomography (CT) revealed a large cystic lesion in the right adnexa (Figure 1B). Therefore, laparoscopic surgery was performed, revealing multiple peritoneal nodules, a few bloody ascites, and a lesion on the right fallopian tube. Hence, peritoneal tumor excision, right salpingo‐oophorectomy, and ascites collection were performed. Cytological analysis of the ascites revealed no malignant cells. However, histopathological analysis of the peritoneal tumor and right fallopian tube revealed STUMPs (Figure 1C). No subsequent events were observed throughout the patient's outpatient follow‐up. However, 7 months postoperation, outpatient sonography revealed left‐upper‐ and right‐lower‐quadrant abdominal masses with complex echogenicity. Therefore, abdominal CT was performed to confirm STUMP recurrence resulting in ileus (Figure 1D). Midline laparotomic tumor excision with IORT (20 Gy/1 fx) and seromuscular layer repair of the descending colonic wall were performed (Figure 1E). Histopathology again confirmed recurrent STUMPs (Figure 1F). After the operation, the patient regularly received outpatient ultrasound and CT scan follow‐up and has remained recurrence‐free for more than 7 years.

**FIGURE 1:**
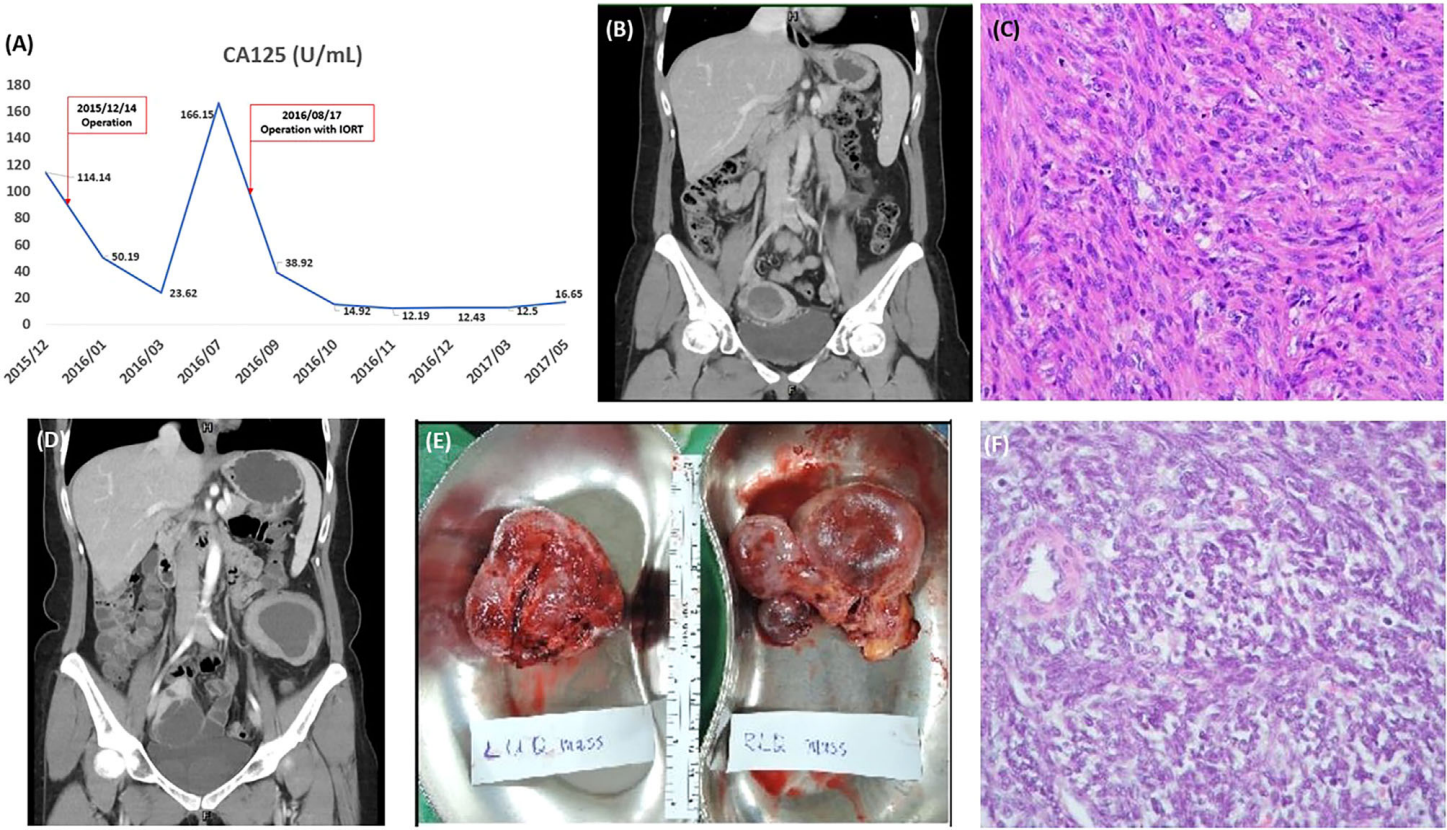
(A) Serum levels of CA‐125 with STUMP recurrence. (B) Large cystic lesion with an enhanced thickened wall in the right adnexa (5.5 cm) observed on CT scan 6 months after the first surgery. (C) The tumor shows spindle cells with mild to moderate atypia and frequent mitosis (up to 20 per 10 high‐power fields) arranged in intersecting and focal herringbone patterns. No apparent tumor necrosis is appreciated. (D) At 7 months after the second surgery, CT scan revealed persistence of recurrent ovarian malignancies in the bilateral pelvis and left peritoneum, with mechanical ileus resulting from ileum fixation and requiring IORT. (E) Left‐upper‐quadrant (LUQ) and right‐lower‐quadrant (RLQ) abdominal masses were eradicated (LUQ: 6.2 × 6.0 × 4.0 cm^3^; RLQ: 8.0 × 4.3 × 3.2 cm^3^). (F) The histopathological changes of the tumor are similar to the previously resected one, except for a lower mitotic rate (up to 7 per 10 high‐power fields).

Although the clinical manifestations of STUMPs are similar to those of leiomyomas, approximately 0%–36% of patients with STUMPs experience recurrence,^2^ with the most common sites of distant recurrence being the lungs and abdomen.^3^ Reports regarding the time interval of STUMP recurrence vary considerably in the literature. A study reported the median interval for STUMP recurrence to be 34 months.^4^ Over the past decade, only seven cases of STUMP recurrence within 1 year following initial mass excision have been documented, with no study reporting patients experiencing recurrence twice within a year. In contrast to radiotherapy, the value of which in recurrent STUMP treatment remains unclear, hormone therapy was reported to be a successful salvage treatment, presumably because of the positive estrogen and progesterone receptors involved in the majority of cases of STUMPs.^5^ Among all published studies, radiotherapy has been used for only three patients, and no studies have used IORT for STUMP recurrence.

To our knowledge, this is the first case report of a patient who experienced rapid STUMP recurrence twice and underwent IORT as a salvage treatment after the second recurrence with disease‐free survival for more than 7 years. Our findings indicate the importance of considering the possibility of rapid STUMP recurrence. They also indicate the potential of IORT as a therapeutic option for STUMP recurrence. Further research is required to validate the clinical efficacy of IORT for STUMP treatment.

## CONFLICT OF INTEREST STATEMENT

All authors declare no conflict of interest.
